# Particleboards with Various Biomass Residues

**DOI:** 10.3390/ma18112632

**Published:** 2025-06-04

**Authors:** Electra Papadopoulou, Dimitrios Moutousidis, Christos Achelonoudis, Stavros Tsompanidis, Christina Kyriakou-Tziamtzi, Konstantinos Chrissafis, Dimitrios N. Bikiaris

**Affiliations:** 1 Chimar Hellas S.A., 15 km National Road Thessaloniki—Polygyros, Thermi, 570 01 Thessaloniki, Greece; dimitris.moutousidis@ari.gr (D.M.); cha@ari.gr (C.A.); 2 PHEE PC, Kolokotroni 17, Rio, 265 04 Patras, Greece; stavros.tsob@gmail.com; 3 School of Physics, Faculty of Sciences, Aristotle University of Thessaloniki, 541 24 Thessaloniki, Greece; chkyriak@physics.auth.gr (C.K.-T.); hrisafis@physics.auth.gr (K.C.); 4 School of Chemistry, Faculty of Sciences, Aristotle University of Thessaloniki, 541 24 Thessaloniki, Greece; dbic@chem.auth.gr

**Keywords:** particleboard, biomass, circular economy, urea-formaldehyde, pMDI

## Abstract

Particleboards were developed by replacing a part of wood with various biomass residues, including coffee bean husks, spent coffee grounds, thistle, *Sideritis* and dead leaves of *Posidonia oceanica*. These materials were analysed to determine their physicochemical properties like the moisture content, pH, and buffer capacity, using standard laboratory techniques, while thermogravimetric analysis (TGA), Fourier-transform infrared spectroscopy (FTIR), and X-ray diffraction (XRD) were also used for their further characterisation. The results revealed that all biomasses contained cellulose, hemicellulose, and lignin in varying proportions, along with differing degrees of crystallinity. To produce particleboards, the biomasses were bonded using two types of adhesives: (a) conventional urea-formaldehyde resin (UF) and (b) polymeric 4,4′-methylene diphenyl isocyanate (pMDI). Laboratory-scale, single-layer particleboards were manufactured simulating industrial production practices. These panels were evaluated for their mechanical and physical properties according to European standards. The findings showed a general reduction in mechanical performance when compared to conventional wood-based panels. However, panels made with coffee grounds and *Posidonia* showed improved resistance to thickness swelling after 24 h in water at 20 °C. Additionally, all experimental panels exhibited lower formaldehyde content than wood-based reference panels. This study demonstrated the feasibility of upcycling biomass residues as a sustainable alternative to virgin wood in the production of particleboard, providing a resource-efficient solution for specific interior applications within a circular economy framework.

## 1. Introduction

Particleboards are common wood composite products that find a variety of applications in interiors, ranging from furniture to decorative cladding. They are typically made by mixing wood particles (chips) with resin—usually in a ratio of about 90:10—and then shaped under high temperature and pressure. However, the rising global demand for high-quality wood-based products has shifted focus toward engineered woods that retain more of wood’s natural form, such as laminated veneer lumber (LVL) and cross-laminated timber (CLT). As a result, the availability of raw wood resources for particleboard manufacturing is becoming increasingly limited.

Agricultural and marine residual biomass has evolved as a promising candidate for the production of bioproducts [1] and for replacing wood in particleboards. Regarding their use in particleboards especially, over the course of the last few years, many scientists have carried out research in this direction, and many kinds of biomass have been tested. Among them, the most highlighted ones are straw (rice, wheat), stalk (cotton), bagasse, seed/fruit, leaf, grass, and palms [2,3].

Cut Rizka Maulida et al. (2021) [4] studied the production of particleboards from coffee ground wastes with different granulometry (20 mesh and 40 mesh) and epoxy resin as a binder. They found that the mechanical properties of such panels depend on the composition and particle size of coffee grounds, while the physical properties (density and thickness swelling) do not change significantly for different particle sizes and compositions. Importantly, the resulting panels met ANSI standards, demonstrating the feasibility of using coffee grounds in particleboard manufacturing.

Mário Vanoli Scatolino et al. (2017) [5] evaluated the feasibility of using coffee parchment at the levels of 0, 10, 20, 30, 40 and 50% in relation to eucalyptus wood, for the production of particleboard. As a binder, a typical urea-formaldehyde (UF) resin was used. Their results showed that up to 10% of coffee parchment could be added without compromising the physical and mechanical performance of the panels.

Nisakorn Nuamsrinuan et al. (2019) [6] investigated the production of particleboard from coffee husks with and without an isocyanate adhesive as a binder at levels from 7 to 13%wt. The panels successfully met the requirements of the TIS.876/2547 standard [7], confirming their structural integrity.

Pornchai Rachtanapun et al. (2012) [8] compared particleboards produced from coffee waste using two different adhesives: UF and polymeric methylene diphenyl diisocyanates (pMDI). The panels were tested for their density, moisture content, thickness swelling, water absorption, bending strength and modulus of elasticity. It was found that particleboards bonded with pMDI were superior to those bonded with UF, while the samples with more than 18% urea–formaldehyde content and all the samples with pMDI adhesive met the Thai industrial standards.

Samson Ayele Bekalo et al. (2010) [9] examined a range of coffee-based substrates (husk, hulls, and wood) combined with various resins (pMDI, Kauramin 534, and Kaurit 390). Based on the results of their mechanical, thermal, and hygric testing, it was found that depending on the type and amount of resin, the particleboards fulfil the requirements of European standards with respect to general use in dry conditions and partially humid environments.

In an effort to explore alternative ways to recycle *Posidonia* natural waste, A. Maciá et al. [10] evaluated biocomposites made from *Posidonia oceanica* (seagrass) and pine wood particles, aiming at the replacement of structural wood in particleboards used in construction [10]. They found that particleboards made using 75% *Posidonia oceanica* and 25% wood particles bonded with 20–30% MDI resin achieved mechanical properties comparable to 100% wood-based panels. This suggested substantial potential for replacing wood with marine biomass while maintaining structural performance.

Sandra Monteiro et al., (2020) [11] prepared low-density panels from cardoon (thistle) fibres bonded with a bio-based binder from starch, chitosan and glycerol. The measured values of density and internal bond strength comply with the standard requirements for general-purpose lightweight particleboards intended for use in dry conditions, as specified by CEN/TS 16368 [12].

Marvin A Batiancela et al. (2014) [13] studied the manufacturing of particleboards using waste tea (*Camellia sinensis*) leaves mixed in various proportions with *Paraserianthes falcataria* (moluccan sau) wood particles. As a bonding material, a commercial liquid urea-formaldehyde resin was used. The results indicated that boards containing 20–50% wood particles met the EN 312-2 (1996) [14] standards for general-purpose particleboards. Notably, boards made solely from tea leaves exhibited low thickness swelling and water absorption, suggesting excellent dimensional stability.

Iwan Risnasari et al. (2019) [15] investigated the replacement of meranti wood (*Shorea* sp.) particles with waste tea leaves (*Camellia sinensis* L.) in the production of particleboards with a typical urea-formaldehyde (UF) resin. They found that such panels had lower overall performance than a conventional particleboard but lower formaldehyde, with the panel made entirely of tea having the lowest levels. The phenolic compounds in tea leaves have been shown to react with formaldehyde, reducing emissions from UF-bonded particleboards.

Although several studies have investigated the use of various tea residues in particleboard production, no literature references were found specifically regarding *Sideritis* biomass. Additionally, the above previous works [4,5,6,8,9,10] involving coffee residues and *Posidonia oceanica* waste often relied on binder contents significantly higher than those typically employed in industrial settings, which limits their practical applicability.

In the present study, dead *Posidonia* leaves, coffee husks, spent coffee grounds, thistle, and *Sideritis* biomass were evaluated as partial replacements for wood in particleboard manufacturing. Prior to their incorporation, these materials were thoroughly characterised in terms of their chemical composition, physical and structural properties, as well as their thermal behaviour. Particleboards were then produced under conditions simulating industrial practice, using binder levels aligned with commercial standards. The mechanical and physical properties of the resulting panels were assessed according to relevant European standards (EN), ensuring their compatibility with current industry requirements.

This study is both timely and essential, as it fills a gap in the literature regarding the use of the studied waste materials under industrial conditions and demonstrates the viability of multiple underutilized biomass types in engineered wood products. By broadening the spectrum of alternative lignocellulosic resources, this research supports the diversification of raw material supply chains and contributes meaningfully to the advancement of sustainable material solutions. The findings offer valuable insights that could guide industrial transitions toward more resilient, environmentally responsible production systems within the framework of the circular economy.

## 2. Materials and Methods

### 2.1. Materials

For the wood-based reference panels, industrial wood particles from pine (*Pinus sylvestris* L.) supplied by the company CHIMAR HELLAS (Thessaloniki, Greece) were used. For the experimental panels, all residual biomasses were provided by the company PHEE (Patras, Greece). Specifically, a quantity of dead *Posidonia oceanica* leaves were collected from local coastal areas, and after cleaning them with fresh water, they were dried at 20–21% moisture content. Fibrous peels from thistle flower peels and stems as well as siderite stems from local farmers were also provided by PHEE company. Τhe leftover materials after processing coffee beans (coffee husks) and making coffee beverages (spent coffee ground) were collected from local companies active in the coffee sector. Regarding the adhesives, pMDI is a bulk material, while a typical urea-formaldehyde resin was prepared and characterised by CHIMAR, as previously reported [16].

### 2.2. Preparation of Materials

The industrial wood particles were sieved to receive fractions of fine (2–3 mm) and coarse (3–4 mm) material. The experimental biomass was used at the dimensions they were provided without further reductions in their size. All materials were dried in an oven at 80 °C to achieve a moisture content of about 3–6% that is needed for their use in the production of particleboards.

### 2.3. Methods for Characterisation of Different Types of Biomasses

The physicochemical properties of these materials (moisture content, pH and acidic buffering capacity) were determined using typical laboratory methods. Each measurement was performed in triplicate. For the study of their composition, structure and thermal performance, instrumental analysis was conducted (FTIR, XRD, TGA).

#### 2.3.1. Moisture Content

The moisture content (MC) of the biomass was determined using the gravimetric method. Approximately 3 g of each sample was weighed, then dried at 103 ± 2 °C until a constant weight was achieved. The MC was calculated using the following formula, expressing moisture content as a percentage of the total weight of the sample (dry wood material plus water) [17,18,19].

$$MC\;\%=\frac{weight\;of\;wet\;sample-weight\;of\;dried\;sample}{weight\;of\;wet\;sample}\times \mathrm{100}$$

#### 2.3.2. pH and Acid Buffering Capacity

A thorough understanding of the pH and buffering capacity of biomasses is crucial for their efficient application in adhesive processes. To measure pH, aqueous extract solutions of the various biomasses were prepared by refluxing 25 g of ground biomass in water at 100 °C for 20 min. Following extraction, the solutions were filtered and stored in sealed Erlenmeyer flasks. All measurements were conducted within 24 h using a digital pH meter (Crison Instruments, S.A., Alella, Spain). To determine the acid buffering capacity (ABC), the samples were then titrated to pH 3 with nominal 0.025 N HCl (for alkalinity determination). The ABC was expressed as ml of HCl consumed after correction for the volume of titrant required to adjust the distilled water to the target pH [20].

#### 2.3.3. Fourier-Transform Infrared (FTIR) Spectroscopy

For the study of biomass via Fourier-transform infrared spectroscopy, it was necessary to produce KBr potassium bromide tablets weighing ≈ 200 mg, with a diameter of ≈13 mm, containing 1% by weight of each biomass. A PERKIN ELMER Spectrum 1000 spectroscope (PERKIN ELMER, Waltham, MA, USA) in the spectral range 4000–400 cm^−1^, with a resolution of 4 cm^−1^ for 32 scans, was used to perform the transmission spectra.

#### 2.3.4. X-Ray Diffraction (XRD) Measurements

The study of crystallinity and the different phases that coexist in biomasses was carried out using the X-ray diffraction technique. All materials were measured in powder form with a particle size less than 200 µm, using the Rigaku Ultima Plus two-cycle diffractometer (Rigaku Holdings Corp, Tokyo, Japan) with Bragg–Brentano geometry and water-cooling system. The analysis was carried out at an energy level of 40 kV, with an electric current of 30 mA, for an angle range from 5° to 60° and a step of 0.05°/1.5 s. The identification of the crystal phases was performed with the contribution of the computer program Jade. In all cases, the radiographs represent the characteristic structure of materials. To determine the crystallinity degree from XRD data, the commonly used Segal method was applied [21]. In this method, crystallinity is calculated by comparing the intensity of the 200 cellulose peaks (Ic), which appear at 2θ = 22–23°, to the intensity at the minimum between the 200 and 1–10 cellulose peaks (Iam), at approximately 2θ = 18°, after background subtraction [22]. According to this, the apparent crystallinity is calculated based on the following equation:

$$CrI\left(\%\right)=\frac{Ic-Iam}{Ic}\times 100$$

#### 2.3.5. Thermogravimetric Analysis (TGA)

The thermal behaviour of the biomasses was studied using the SETARAM SETSYS TG-DTA 16/18 instrument (Setaram, Lyon, France). A small quantity of all materials was first pulverized to a particle size less than 200 µm and then tested for their thermal decomposition from ambient temperature up to 1200 °C, at a heating rate of 10 °C/min, in an oxidizing atmosphere, while the initial mass used was in the order of 3 ± 0.4 mg.

### 2.4. Preparation of Particleboards

Single-layer particleboards were produced using a computer-controlled laboratory hot press. For their manufacturing, fine and coarse wood particles were mixed at a ratio of fine/coarse particles = 60/40, while for the experimental panels, 30% of wood fine particles were replaced by the various residual biomasses. The production process followed was a simulation of the industrial practice [23]. All materials, after drying, were mixed with the binder (pMDI or a mixture of resin/water/hardener in the case of the UF resin). The adhesive was sprayed on the particles at a level of 3–9% on dry biomass. The materials were mixed in the blending machine for about 1 min, and then they were formed in a mat with dimensions 50 cm × 50 cm and 13 cm thick. The mat was then cold pressed followed by hot pressing (15–35 kg/cm^2^ for particleboards with a density of 0.4–0.8 g/cm^3^) at temperatures of 150–200 °C. The pressing time is proportional to the thickness of the particleboard and is approximately 0.3–0.4 min per millimetre of finished product thickness. The final density of the panels was 700 kg/m^3^.

### 2.5. Particleboard Performance Tests

After pressing, the successful panels were cooled to room temperature and conditioned in a controlled climate (20 ± 2 °C and 65 ± 5% relative humidity) for 24 h to stabilize their moisture content prior to testing. The panels were then trimmed and cut into test specimens in accordance with the requirements of the relevant European standards. To evaluate their mechanical performance, three key properties were assessed: internal bond (IB), modulus of rupture (MOR), and modulus of elasticity (MOE). IB, which reflects the tensile strength perpendicular to the panel surface and indicates adhesive performance, was measured according to EN 319 [24]. MOR and MOE, which represent bending strength and stiffness, respectively—key indicators of structural performance—were determined following EN 310 [25]. Dimensional stability was assessed by measuring thickness swelling (TS) after 24 h of water immersion, as per EN 317 [26], which is critical for applications exposed to moisture. Finally, the formaldehyde content, (FC) at 6.5% Moisture Content (MC) a crucial parameter for assessing environmental safety and compliance with emission regulations, was determined using the perforator method according to EN 120 [27].

## 3. Results and Discussion

This study aims to evaluate the efficiency of replacing virgin wood with residual biomass, both as a sustainable alternative in the event of wood shortages and as a means of adding value to residual materials. The test results are presented below.

### 3.1. Properties of the Urea-Formaldehyde Reins

The properties of UF resin used for the binding of the various biomasses are cited below in Table 1.

### 3.2. Properties of Studying Biomaterials

The properties of all biomasses determined with typical laboratory analysis are shown in Table 2.

The results show that all materials have a more or less acidic pH, with *Sideritis* being the most acidic one among the experimental materials. Their biggest difference compared to wood is their acidic buffer capacity, which is significantly higher than that of wood. These variations might be related to differences in their chemical composition, particularly in their ash content, mineral profile, and organic compounds, as well as their distribution in the biomass [20].

### 3.3. FTIR Analysis Results

**Figure 1:**
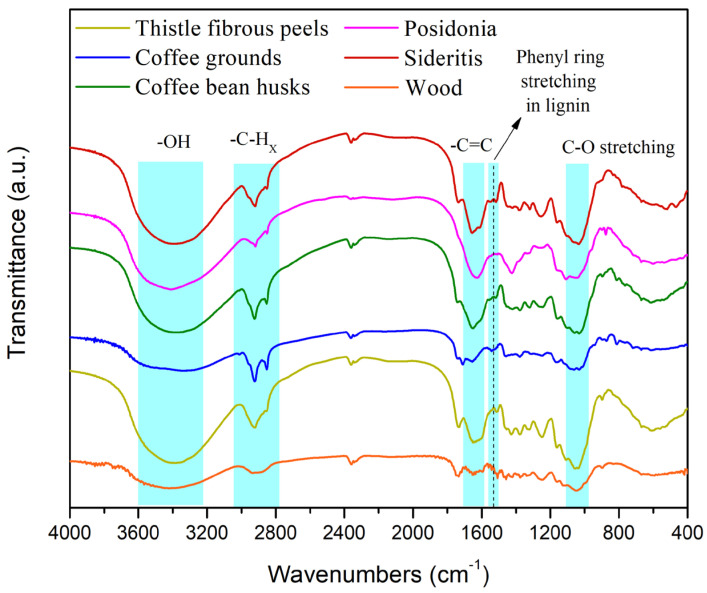
FTIR spectra of various types of biomass.

**Figure 2:**
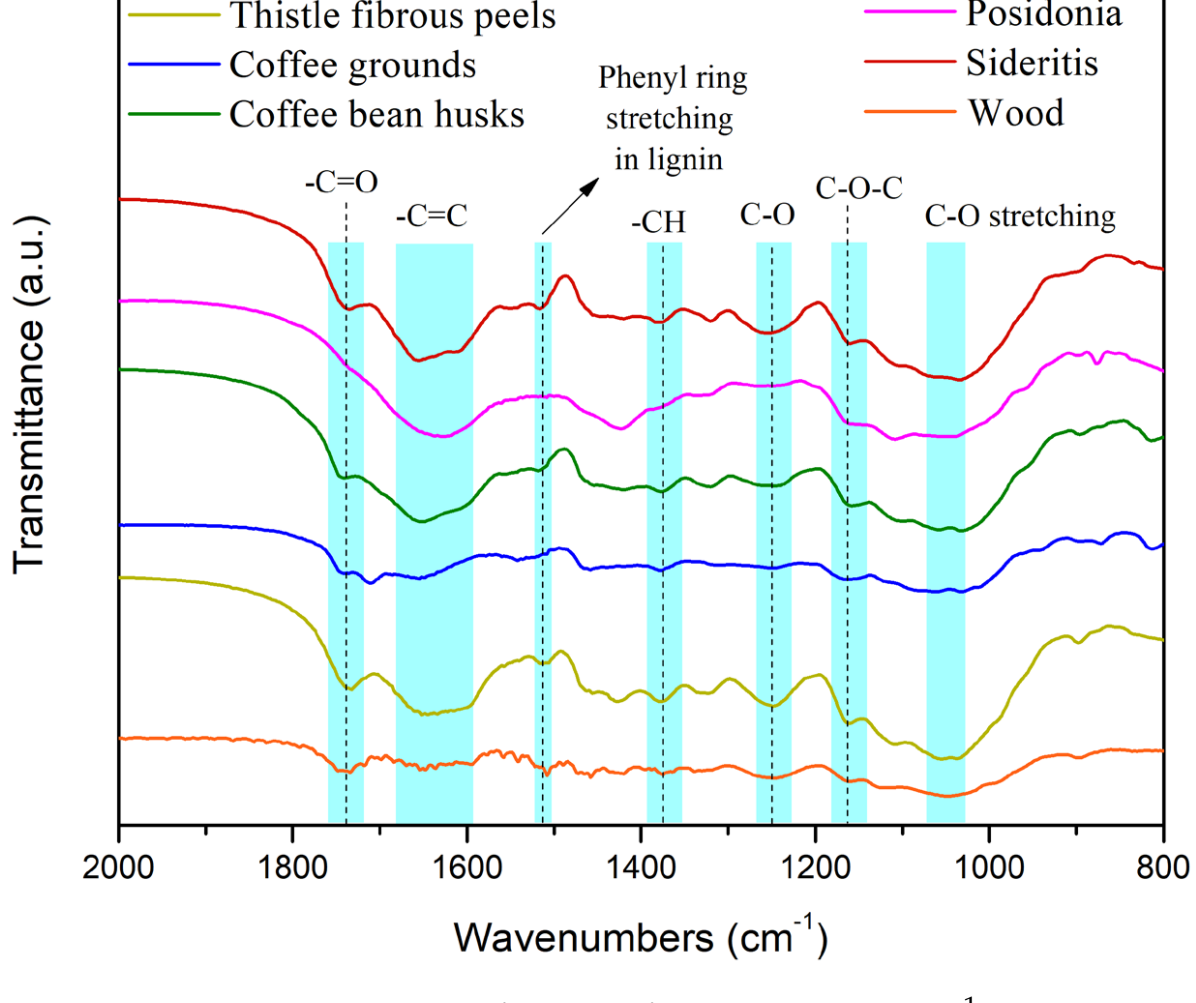
FTIR spectra—magnification of area 2000–800 cm^−1^.

Figure 1 and Figure 2 show the FTIR spectra collected from the various types of experimental biomass.

In Figure 1, the broad band between 3600 and 3200 cm^−1^ is attributed to OH vibrations, while the twin peaks at about 2900 cm^−1^ and 2800 cm^−1^ are due to asymmetrical and symmetrical stretching of aliphatic C–H usually displayed in fatty acids [28,29].

The enlargement of the graph at 2000–800 cm^−1^ shows a variety of peaks for each material.

At the 1730–1740 cm^−1^ band, which is preferentially ascribed to C=O groups of acids, aldehydes or ketones, *Posidonia* has no peak, while all other materials have, meaning that compounds without carbonyl groups exist in this material.

The 1680–1580 cm^−1^ band is attributed to C=C stretching. At higher wavenumbers, there are aliphatic C=C bonds (alkenes), and at lower wavenumbers, there are aromatic C=C bonds. Hence, we can see that *Posidonia* has only aromatic C=C bonds (one peak at 1628 cm^−1^), while the coffee ground sample seems to have no aromatic C=C bonds. All other materials have both types of C=C bonds at different ratios. Also, the wavenumbers at around 1650 cm^−1^ are attributed to caffeine molecules. According to the graph, we can see that coffee husks have a more intense peak in this area than waste coffee grounds, meaning that, most probably, coffee husks contain more caffeine than waste coffee grounds.

The band between 1510 and 1550 cm^−1^ is possibly ascribed to conjugated C=N systems and amino functionalities (N–H of amide II). *Posidonia* does not form such a peak in this area. Moreover, the peak located at 1384 cm^−1^ is assigned to C–H deformation in cellulose and hemicellulose, the peak at 1243 cm^−1^ is associated with C–O stretching vibrations in hemicellulose and lignin, while the peak at approximately 1158 cm^−1^ has been found to relate with C–O–C stretching in cellulose. Finally, the region at around 1060 cm^−1^ appears due to C–O stretching in cellulose and hemicellulose and C–C stretching in cellulose, as well [30]. Based on the study of [31], the following can be stated:

- Cellulose is rich in compounds with O–H and C–O bonds;
- Hemicelluloses contain more C=O compounds;
- Lignin might be rich in methoxyl—OCH_3_, C–O–C and C=C (aromatic ring)-containing compounds.

We can say that all materials contain cellulose, hemicelluloses and lignin but at different ratios. Comparing their spectra, we can say the following:

- All materials have fatty acids to some extent;
- Coffee grounds have less cellulose than other materials (considering that cellulose gives any crystallinity to the material, this result is also confirmed by the XRD study below where the coffee ground sample has the lowest crystallinity);
- *Posidonia* has less hemicelluloses than the others (in accordance with [32,33]);
- Lignin’s contribution is more pronounced for *Sideritis* and thistle (intense peak at 1516 cm^−1^ representing phenyl ring stretching in lignin structure [30,34]).

### 3.4. XRD Analysis

**Figure 3:**
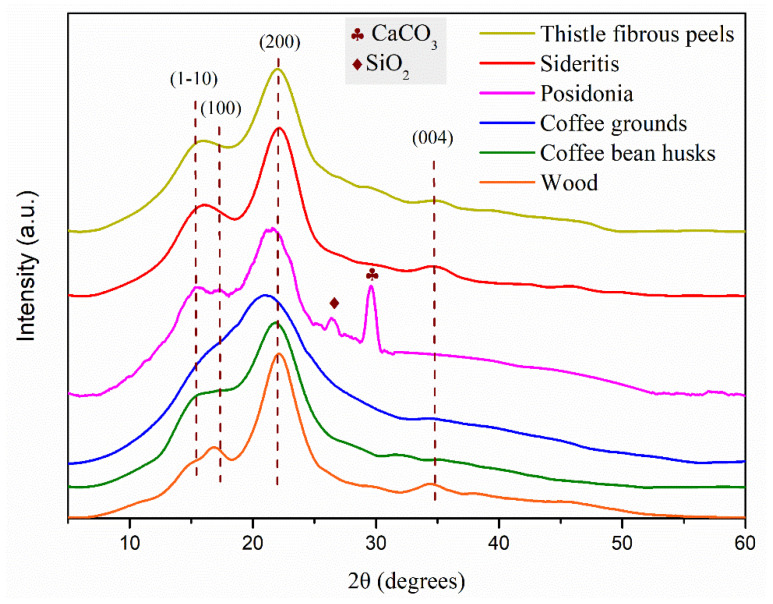
X-ray diffraction (XRD) study results of PHEE samples.

Figure 3 shows the results of the XRD analysis

The X-ray diffraction (XRD) patterns of lignocellulosic biomass, as depicted in Figure 3, feature broad and overlapping peaks that reflect the semi-crystalline nature of their main structural components: cellulose, hemicellulose, and lignin. The most prominent contribution comes from cellulose, particularly the crystalline regions, which generate distinct peaks around 2θ ≈ 16° and 2θ ≈ 22°, characteristic of the (1-10), (100), and (200) crystallographic planes of cellulose I, the naturally occurring form of cellulose in plant biomass. Hemicellulose, being mostly amorphous, does not contribute significantly to sharp diffraction peaks but adds to the background hump, while lignin, another largely amorphous component, contributes to the overall broadness of the pattern and to background scattering rather than sharp peaks. The additional sharp peaks, one at around 29° and another at approximately 27°, correspond to crystalline impurities and, more specifically, to calcium carbonate (CaCO_3_) and silica (SiO_2_), respectively [35].

When comparing the XRD patterns of the different biomass samples, noticeable variations reflect their distinct biochemical compositions. Wood, *Sideritis* and thistle fibrous peels exhibit sharp and intense peaks at 2θ ≈ 22°, implying higher cellulose crystallinity. Wood, especially, exhibits the highest crystallinity among all the investigated samples, as observed in Table 3, resulting from its structural role in plants, where cellulose microfibrils are highly ordered to provide mechanical strength. High values of crystallinity have also been detected in the literature, like Aspen and Spruce pulp, found to have around a 75% crystallinity index [36]. In contrast, coffee bean husks and *Posidonia* display broader and less intense peaks, indicating a more amorphous structure and possibly a higher content of lignin and/or hemicellulose. *Posidonia* also stands out, with distinct peaks at 27° and 29°, attributed to the mineral content (calcium carbonate and silica), typical of its marine origin. Coffee grounds present the most amorphous XRD pattern, compared to the other lignocellulosic materials, which is expected considering that the as-received and measured sample has already been subjected to grinding in order to produce a coffee beverage. Grinding generally reduces crystallinity, since mechanical forces disrupt the ordered cellulose microfibrils, breaking hydrogen bonds and converting crystalline regions into amorphous ones. This amorphization is particularly noticeable in XRD patterns, where peak intensity diminishes and broadens, reflecting a loss of structural order [37]. Yanjuan Zhang et al. found that the mechanical activation of sugarcane bagasse resulted in decreased crystallinity [38]. More specifically, the untreated sample was characterised by a crystallinity degree of approximately 52%, while the same value for the mechanically treated specimen was 22%. The degree of crystallinity for all investigated lignocellulosic samples is depicted in Table 3.

### 3.5. TGA Study Results

**Figure 4:**
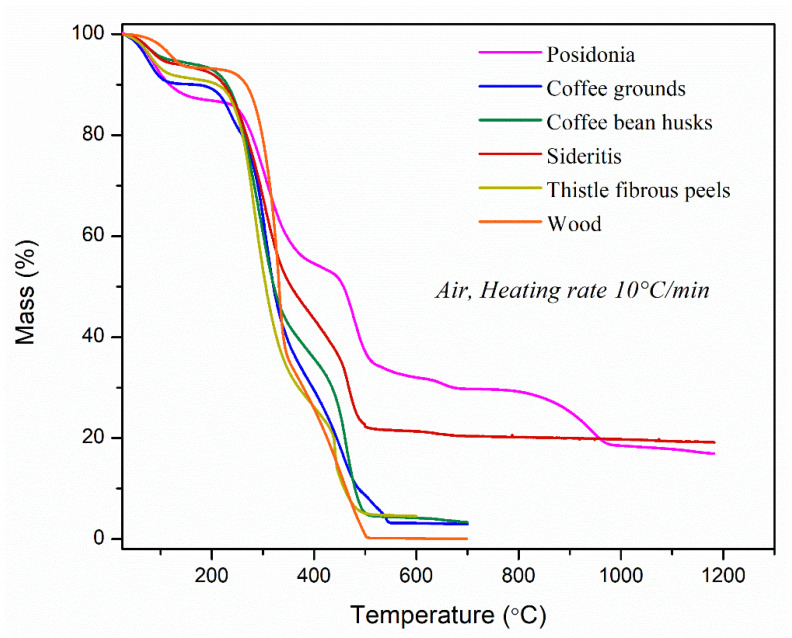
Thermogravimetric analysis (TGA) results.

The temperature ranges for the TG tests were adjusted based on the specific thermal degradation profiles of each biomass material. Agricultural residues such as thistle and *Sideritis* decompose at slightly lower temperatures than marine biomass such as *Posidonia oceanica*, which contains more thermally stable inorganic materials. The temperature ranges were selected to fully capture the decomposition stages of each sample. Figure 4 shows the TGA curves of mass loss for the various experimental materials.

The decomposition steps observed in the TGA graph reflect the oxidative thermal degradation of the biomass samples. The first stage, typically occurring below 200 °C, corresponds to the loss of physically bound moisture. The second stage, usually between 200 °C and 400 °C, involves the oxidative decomposition of hemicellulose and cellulose. These components react with oxygen, leading to accelerated weight loss and the formation of volatiles and char [39]. Hemicellulose, being less thermally stable, decomposes earlier, followed by cellulose. The third stage, extending from around 400 °C to 600 °C or higher, is mainly attributed to the oxidation of lignin and the residual carbon-rich char formed in earlier stages. Above 600 °C, the rate of mass loss significantly decreases, indicating the combustion of remaining char and the approach to complete burnout, leaving behind ash.

When comparing the thermal degradation profiles of the samples, noticeable differences emerge in both the onset and extent of decomposition. *Posidonia* leaves and *Sideritis* exhibit greater thermal resistance, with slower and more gradual weight loss, possibly suggesting a higher lignin or fixed carbon content that resists oxidative attack. In contrast, wood, coffee grounds, coffee bean husks and thistle degrade earlier and more sharply, indicating lower thermal stability and potentially higher levels of easily oxidizable components. The notable mass loss observed in *Posidonia* leaves between 600 °C and 750 °C can be attributed to the decomposition of carbonates, primarily calcite (CaCO_3_). This is also confirmed by the sample analysis using X-ray diffraction (XRD) and supported by literature data, which indicate that calcite undergoes thermal decomposition above 600 °C [40]. The significant mass loss observed between 900 °C and 1000 °C in the *Posidonia* specimen can be attributed to the decomposition of inorganic constituents, particularly sodium chloride (NaCl), which is typically present in high concentrations due to the marine origin of the plant. While NaCl itself has a high melting point (around 800 °C) [41] and is thermally stable under normal conditions, in the presence of oxygen and other mineral components, it can participate in high-temperature reactions. Around this temperature range, NaCl may begin to volatilize or interact with other inorganic materials (such as silicates or sulfates), leading to the formation of gaseous species or more volatile compounds. Additionally, some chlorides can catalyse the oxidation or gasification of residual carbon, further contributing to mass loss [42,43]. Overall, the study with thermogravimetric analysis (TGA) showed that *Posidonia* is the most thermally stable material, followed by *Sideritis*. The mass loss (%) and temperature range of all investigated lignocellulosic samples throughout the distinct thermal degradation stages are summarized in Table 4.

### 3.6. Properties of Particleboards

All experimental materials were used as a 30% wood substitute in the production of particleboards, using, as a binder, (a) a typical UF resin and (b) pMDI.

**Figure 5:**
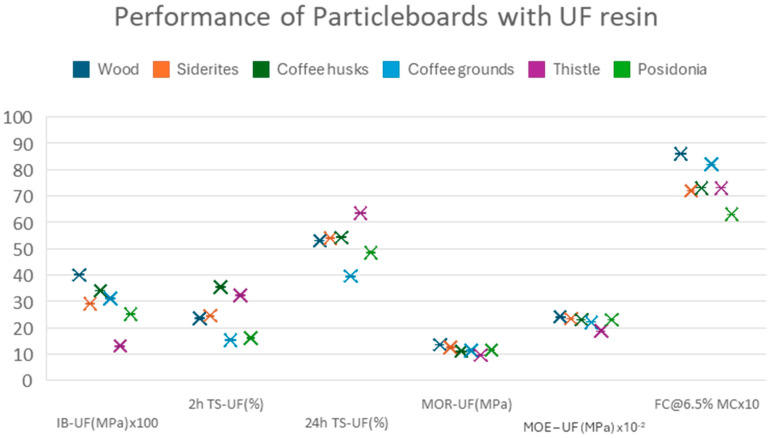
Performance of particleboards with UF resin.

The panels were tested for their mechanical and physical properties in accordance with the relevant EN standards. The results, shown in Figure 5, represent the average of six measurements.

The results show a decrease in the mechanical properties of the experimental panels. Coffee grounds and *Posidonia* improve thickness swelling but not the other materials. The formaldehyde content is lower than the control in all cases, with coffee grounds having the smallest difference from the reference.

**Figure 6:**
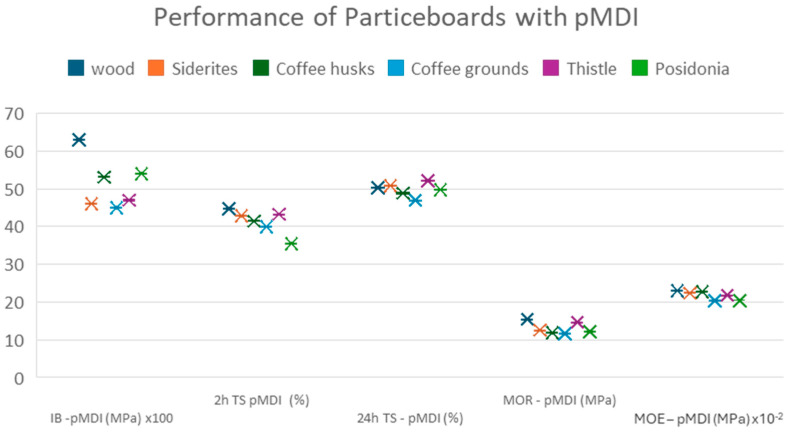
Performance of particleboards with pMDI resin.

Additionally, the various biomasses were used in the production of particleboards with the same process as before but using pMDI as a binder. The results of their performance tests according to European standards are shown in Figure 6.

The results indicate that all experimental panels exhibit lower mechanical properties compared to the reference panels, with the most significant difference observed in internal bond strength. However, their modulus of elasticity (MOE) is comparable, with coffee husks demonstrating the best performance in this regard. In terms of thickness swelling, most experimental panels outperform the control, suggesting a higher resistance to humid environments. Among them, coffee grounds showed the best performance in this property. Although produced on a pilot scale, the results suggest that similar industrial panels could be classified in technical class P1 or P2 (EN312:2010) [44] and be suitable for indoor use in dry environments or as furniture.

**Figure 7:**
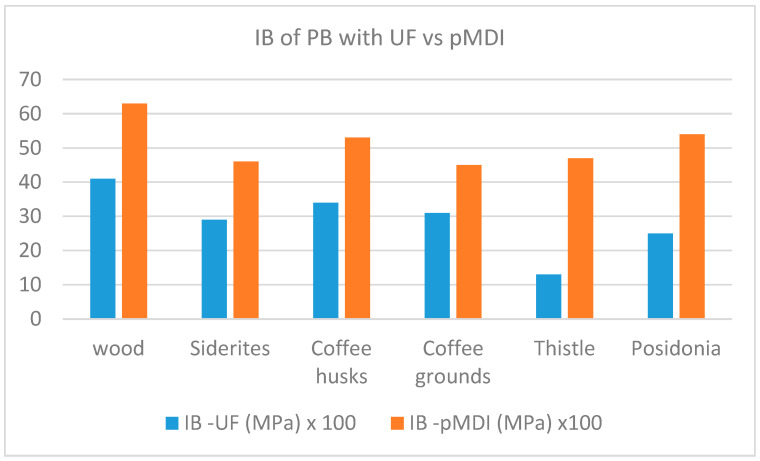
Comparative internal bond performance of particleboards manufactured with pMDI and UF resins.

**Figure 8:**
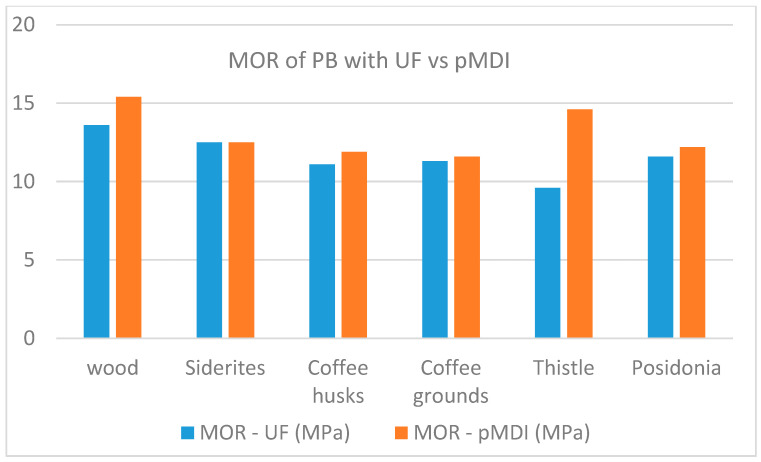
Comparative modulus of rupture performance of particleboards manufactured with pMDI and UF resins.

**Figure 9:**
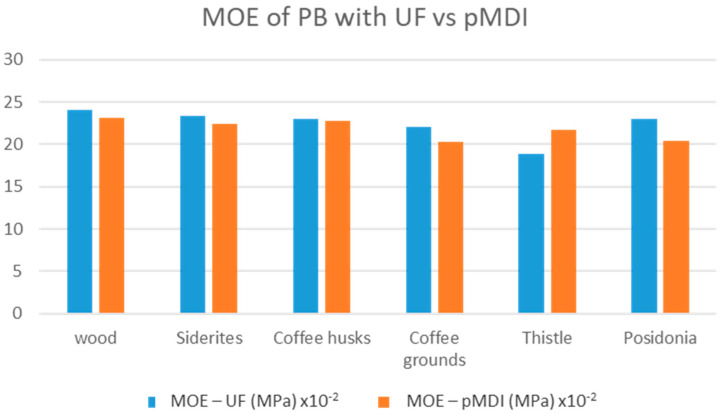
Comparative modulus of elasticity performance of particleboards manufactured with pMDI and UF resins.

**Figure 10:**
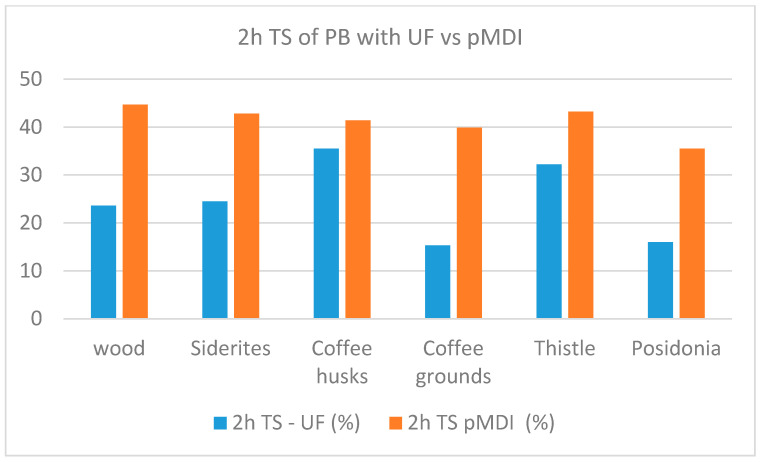
Comparative 2 h thickness swelling performance of particleboards manufactured with pMDI and UF resins.

**Figure 11:**
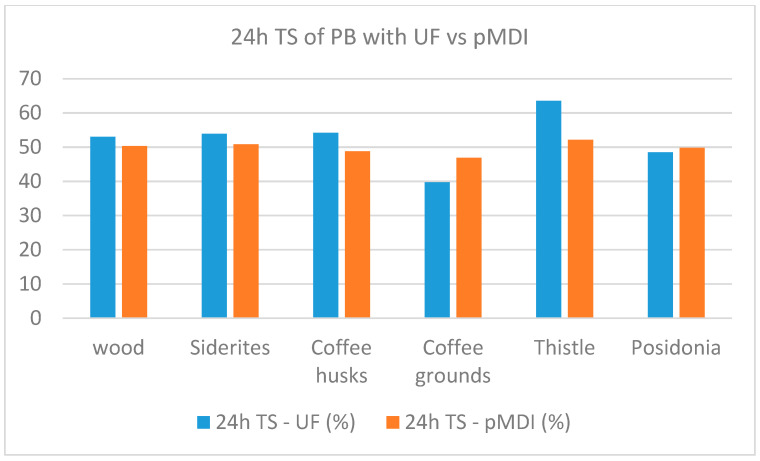
Comparative 24 h thickness swelling performance of particleboards manufactured with pMDI and UF resins.

A comparison of the performance of panels per property when using UF resin and pMDI as binders is shown in Figure 7, Figure 8, Figure 9, Figure 10 and Figure 11.

The comparative study indicates that pMDI produces panels with superior performance in both the internal bond (IB) and modulus of rupture (MOR). In terms of elasticity, however, UF resin results in slightly better performance across all biomass types, except thistle. Regarding moisture resistance, panels bonded with UF resin showed better thickness swelling (TS) performance after 2 h of water immersion. However, after 24 h, pMDI-bonded panels outperformed UF panels when wood was partially replaced with *Sideritis*, coffee husks, or thistle. For panels containing coffee grounds and Posidonia, thickness swelling was lower when pMDI was used as the binder.

## 4. Conclusions

This study is a comprehensive evaluation of the physicochemical, thermal, and structural properties of particleboards made with various residual biomasses aiming to understand how each biomass might influence the final properties of the composite panels. The findings offer important insights into how specific material characteristics affect particleboard performance and highlight pathways for optimizing formulations for targeted applications.

The physicochemical parameters—moisture content, pH, and buffer capacity—are critical in assessing the compatibility between biomass and adhesives. Notably, the buffer capacity varied widely among the biomass types, with coffee bean husks and Sideritis exhibiting the highest values (60 mL and 33 mL, respectively), compared to wood (6 mL). A higher buffer capacity indicates a stronger resistance to pH change during resin curing, which can negatively affect adhesive polymerization, especially for pH-sensitive resins such as urea-formaldehyde (UF). This partly explains the reduced internal bond (IB) strength observed in particleboards incorporating these biomasses.

Thermogravimetric analysis (TGA) revealed that Posidonia and Sideritis have superior thermal stability, with decomposition stages extending to 1200 °C and higher residual ash content (17–19%). This high thermal stability is advantageous for panel performance under temperature variations or exposure to heat during processing. In contrast, coffee grounds and thistle exhibited rapid degradation and lower residual mass, indicating lower fixed carbon and possibly higher volatile content, traits that could reduce the boards’ dimensional and mechanical stability under thermal stress. The strong correlation between buffer capacity and thermal stability is especially relevant. For example, Sideritis, which had a high buffer capacity, also showed elevated thermal resistance. This suggests the presence of chemically resistant compounds (e.g., polyphenolics or high lignin content), which both buffer acidic environments and resist oxidative degradation, valuable traits for enhancing board longevity in high-moisture or high-temperature settings.

The FTIR analysis provided deeper insight into the chemical composition of the biomasses, particularly in terms of lignin, cellulose, and hemicellulose content. Coffee grounds exhibited the lowest intensity in the cellulose-associated O–H and C–O peaks, corroborating the low crystallinity values from XRD (45.7%) and suggesting limited structural rigidity. This explains their relatively poor mechanical performance in bending tests but also their superior water resistance, as amorphous regions tend to absorb and desorb water more evenly. On the other hand, Sideritis and thistle demonstrated stronger signals for aromatic skeletal vibrations at ~1600 cm^−1^ and ~1500 cm^−1^, indicating higher lignin content. Lignin-rich biomass is known to provide intrinsic water repellence and thermal stability, which aligns with the good performance of these biomasses in long-term swelling resistance and thermal decomposition studies. Posidonia lacked significant C=O or C=N bands in FTIR spectra, indicating low concentrations of aldehydes or protein-like compounds, but showed substantial aromatic content and mineral-rich composition (confirmed via XRD), which contributed to its remarkable thermal stability and moisture resistance despite lower cellulose and hemicellulose content.

Overall, this study demonstrates that particleboards can be successfully manufactured using various residual biomasses as partial replacements for wood, employing either pMDI or conventional UF resin as a binder. While these panels generally exhibit lower mechanical properties compared to traditional particleboards, they offer significantly reduced formaldehyde emissions (when UF resin is used) and exhibit acceptable tolerance to humid environments. Considering the material selection based on the results, the following can be said:

Coffee Grounds: Low cellulose and high amorphous content make them less suitable for mechanical strength applications but ideal for boards requiring dimensional stability and low formaldehyde emissions.

Posidonia: High thermal resistance and mineral content yield excellent moisture resistance, making it promising for humid environments or coastal applications.

Sideritis and Thistle: Balanced cellulose–lignin composition and high buffer capacity suggest potential for performance enhancements through adhesive optimization.

Coffee Bean Husks: Despite good cellulose crystallinity, their high buffer capacity may inhibit resin curing, requiring either resin modification or pH adjustment strategies.

This study demonstrates that the successful integration of agricultural and marine biomasses into particleboard production depends on a nuanced understanding of each biomass’s physicochemical profile. While the mechanical performance is generally reduced compared to traditional wood-based boards, targeted improvements in formulation, such as resin selection and surface treatment, could overcome these limitations. Residual biomasses like spent coffee grounds and Posidonia offer viable low-emission, moisture-resistant alternatives for non-structural indoor applications, advancing sustainability in wood composite manufacturing.

Future work should explore synergistic combinations of different biomasses, binder modifications (e.g., bio-based or pH-tolerant adhesives), and pre-treatment methods to enhance compatibility and performance. The findings underscore the broader potential of residual biomass utilization in circular economy strategies, reducing reliance on virgin wood while creating value from organic waste streams.

## Figures and Tables

**Table 1:** Properties of UF resin.

Type of Resin	UF
Solids, %	65.02
pH at 25 °C, [ ]	7.91
Viscosity at 25 °C, cP	250
Water Tolerance, mL/mL	1/4
Gel time at 100 °C, s	55.00
Buffer Capacity, %	9.0

**Table 2:** Physical properties of various biomasses.

Name of Material	Moisture Content, %	pH, [ ]	Buffer Capacity, mL
*Sideritis*	11.74	5.34	33
Coffee bean husks	6.18	5.60	60
Coffee grounds	19.25	5.70	15
Thistle fibrous peels	14.84	6.11	23
*Posidonia*	20.81	7.38	16
wood	12.50	5.32	6

**Table 3:** Crystallinity of experimental biomasses.

Material	Degree of Crystallinity
Wood	64.7%
*Sideritis*	62.4%
Thistle	57.8%
Coffee husks	53.5%
*Posidonia leaves*	48.8%
Spent coffee grounds	45.7%

**Table 4:** Summary of TGA results for all investigated biomass samples under air.

Sample	Dehydration Stage		Devolatilization		Char and Mineral Combustion		Ash (Residual Mass %)
	T-Range (°C)	Mass (%)	T-Range (°C)	Mass (%)	T-Range (°C)	Mass (%)	
Wood	25–190	7	190–375	62	375–700	30.95	0.05
*Sideritis*	25–160	7	160–378	46	378–1200	27.8	19.2
Thistle	25–160	9	160–385	63	385–600	23.4	4.6
Coffee bean husks	25–155	6	155–386	56	386–700	34.6	3.4
Coffee grounds	25–150	10	150–387	58	387–700	29	3
*Posidonia*	25–200	13	200–406	33	406–1200	37	17

## Data Availability

The original contributions presented in this study are included in the article. Further inquiries can be directed to the corresponding author.
